# Principles of *N*-Linked Glycosylation Variations of IgG-Based Therapeutics: Pharmacokinetic and Functional Considerations

**DOI:** 10.3390/antib9020022

**Published:** 2020-06-10

**Authors:** Souad Boune, Peisheng Hu, Alan L. Epstein, Leslie A. Khawli

**Affiliations:** Department of Pathology, Keck School of Medicine, University of Southern California, Los Angeles, CA 90089, USA; so3ad86@gmail.com (S.B.); peisheng@usc.edu (P.H.); aepstein@usc.edu (A.L.E.)

**Keywords:** glycosylation, post-translational modifications, pharmacokinetics, effector functions, antibody-dependent cell-mediated cytotoxicity, complement-dependent cytotoxicity, immunogenicity, pharmacodynamics, glycoengineering, antibody-drug conjugates

## Abstract

The development of recombinant therapeutic proteins has been a major revolution in modern medicine. Therapeutic-based monoclonal antibodies (mAbs) are growing rapidly, providing a potential class of human pharmaceuticals that can improve the management of cancer, autoimmune diseases, and other conditions. Most mAbs are typically of the immunoglobulin G (IgG) subclass, and they are glycosylated at the conserved asparagine position 297 (Asn-297) in the CH2 domain of the Fc region. Post-translational modifications here account for the observed high heterogeneity of glycoforms that may or not impact the stability, pharmacokinetics (PK), efficacy, and immunogenicity of mAbs. These modifications are also critical for the Fc receptor binding, and consequently, key antibody effector functions including antibody-dependent cell-mediated cytotoxicity (ADCC) and complement-dependent cytotoxicity (CDC). Moreover, mAbs produced in non-human cells express oligosaccharides that are not normally found in serum IgGs might lead to immunogenicity issues when administered to patients. This review summarizes our understanding of the terminal sugar residues, such as mannose, sialic acids, fucose, or galactose, which influence therapeutic mAbs either positively or negatively in this regard. This review also discusses mannosylation, which has significant undesirable effects on the PK of glycoproteins, causing a decreased mAbs’ half-life. Moreover, terminal galactose residues can enhance CDC activities and Fc–C1q interactions, and core fucose can decrease ADCC and Fc–FcγRs binding. To optimize the therapeutic use of mAbs, glycoengineering strategies are used to reduce glyco-heterogeneity of mAbs, increase their safety profile, and improve the therapeutic efficacy of these important reagents.

## 1. Introduction

Monoclonal antibody (mAb)-based therapeutics have been increasingly studied and utilized as therapeutic agents for the past 20 years [1]. Even though mAb technology was invented early in 1975 by Milstein and Koehler [2], the potential of these agents was not appreciated originally because of anti-drug antibody (ADA) responses in humans induced by murine antibodies [3]. However, with the rapid growth of biotechnology-derived techniques and the advanced knowledge of the immune system, scientists have realized the roll that mAbs can play in the treatment of many diseases [4]. Today, there are more than 60 products of therapeutic monoclonal antibodies (mAbs) that are approved in the US for human use, about 240 in clinical testing, and around 40 entering clinical trials each year [5,6].

**Figure 1:**
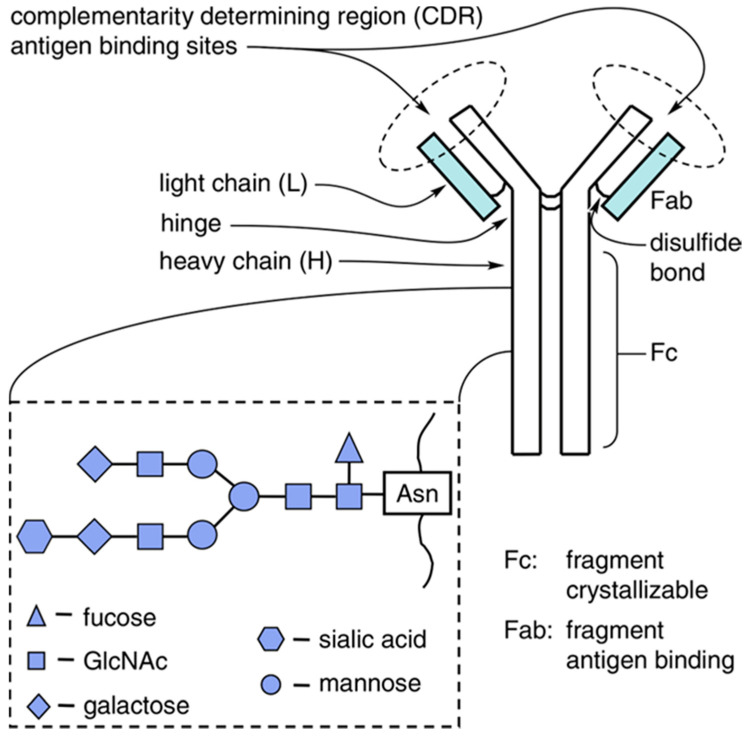
Simplified structure of an immunoglobulin (IgG). Inset shows an example of an IgG Fc diantennary oligosaccharide, which in normal IgG, is attached at an asparagine residue at position 297 (Asn-297). Generally, the oligosaccharide has a core pentasaccharide with varying addition of galactose, fucose, sialic acid, and *N*-acetylglucosamine (GlcNAc). Reproduced from Bakhtiar, 2012 [4] with permission of the copyright owner.

Therapeutic antibodies are generally IgGs. An IgG is a glycoprotein that contains four polypeptide chains: Two identical heavy chains (H) and two identical light chains (L). The light and heavy chains pair by covalent disulfide bonds and noncovalent associations (Figure 1) [4]. Each heavy chain is connected to one light chain by one disulfide bond. Each antibody molecule is made of three globular domain structures forming a “Y” shape, two of which are the fragments that bind to the antigens (Fab) and the other is the fragment crystallizable (Fc) for the activation of Fcγ receptors (FcγRs) on leukocytes and the C1 component of complement [6]. IgG molecules bear *N*-glycosylation at the conserved asparagine at position 297 (Asn-297) in the heavy chain of the CH2 constant domain of the Fc region [6]. The oligosaccharide is an essential player in Fc effector functions including antibody-dependent cellular cytotoxicity (ADCC) and complement-dependent cytotoxicity (CDC), which are major mechanisms of action of therapeutic antibodies located in the Fc region. Alteration of glycan compositions and structures can impact the effector function by causing conformational changes of the Fc domain, which would affect binding affinity to Fcγ receptors [3,5]. Thus, engineering of Fc glycosylation to develop therapeutic monoclonal antibodies with desired characteristics is a promising strategy to enhance functionality and efficacy of therapeutic IgG antibodies. In this review, Fc *N*-glycan structure and biosynthesis are briefly reviewed, followed by a discussion of the knowledge acquired recently about the influence of glycosylation of antibodies on therapeutic antibody immunogenicity, pharmacokinetics (PK), and effector functions. Furthermore, current Fc glycoengineering strategies used to produce mAbs with higher homogeneity and effector functions are introduced and discussed. In the following sections we will also discuss those aspects of glycosylation variations which relate to the PK and pharmacodynamic (PD) parameters of currently approved antibody-based therapeutics.

## 2. IgG Glycan Structure and Biosynthesis

Post-translational modification is a biological process that involves the modification of an amino acid side chain, terminal amino, or carboxyl group by means of covalent or enzymatic modifications following IgG biosynthesis. Generally, these modifications may include phosphorylation, acetylation, glycosylation, sialylation of one or more amino acids in the protein, and also may include the formation of S-S bridges between 2 SH groups on amino acids, and proteolysis. Post-translational modifications contribute to the final tertiary (three-dimensional) structure of IgGs and play a key role in the biological activity and interaction with other cellular molecules such as proteins, nucleic acids, lipids, and cofactors. These modifications are not predictable by the sequence of IgG and are often critical in determining the way IgG behaves (e.g., its function and degradation). Therefore, each therapeutic protein will have a unique post-translational modification profile in its natural state, and as discussed further in this review, the post-translational modification profile of an IgG can potentially impact drug stability, safety, and efficacy.

### 2.1. IgG Glycan Structure

**Figure 2:**
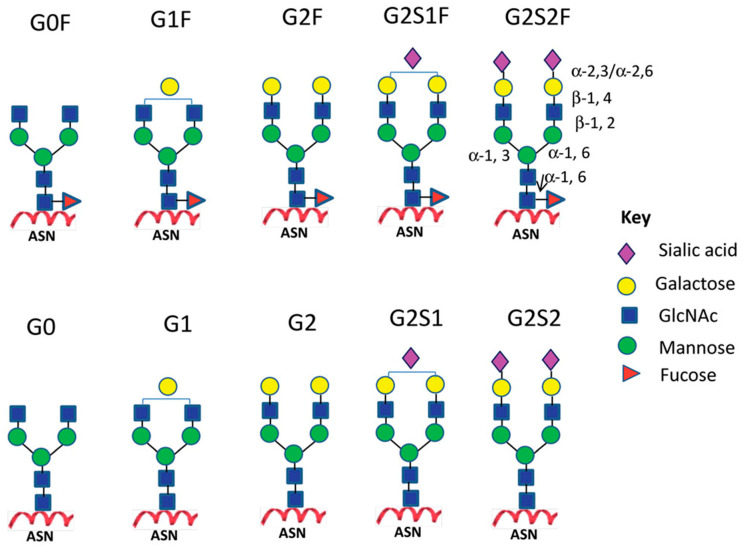
Major *N*-linked glycoforms of therapeutic monoclonal antibodies (mAbs). Reproduced from Liu, 2015 [3] with permission of the copyright owner.

Structurally, the *N*-linked glycans of human IgGs are typically biantennary complexes. Different residues, such as fucose, bisecting GlcNAc, galactose, and sialic acid, can be added to this core biantennary complex structure (GlcNAc2Man3GlcNAc2), generating heterogeneity of the IgG-Fc glycans of normal polyclonal IgGs [5,7]. The heterogeneous glycans can be classified into three sets (G0, G1, and G2), depending on the number of galactose residues in the outer arms of biantennary glycans. Within each of these sets, there are different species that arise from the presence or absence of core fucose and bisecting GlcNAc (Figure 2) [3].

### 2.2. Glycan Biosynthesis in Human Cells

**Figure 3:**
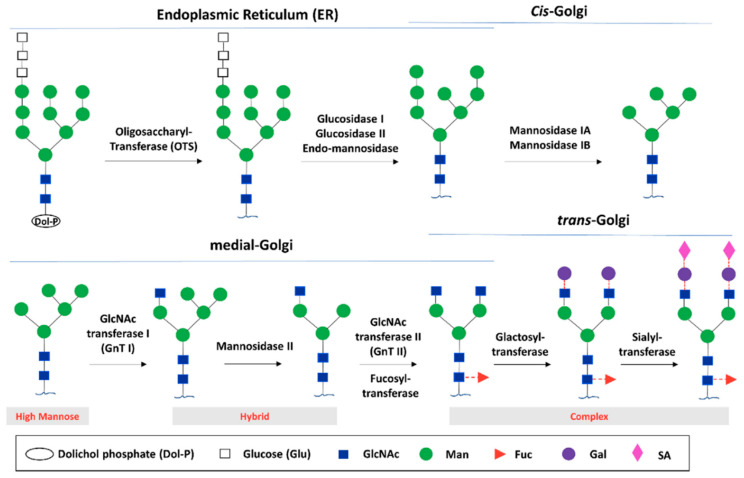
Glycan biosynthesis through the endoplasmic reticulum (ER) and Golgi glycosylation pathways. The biosynthesis begins with the processing of the initial high mannose *N*-glycan in the ER, followed by transferring into the cis-Golgi to generate the core *N*-glycan substrate used for further diversification in the trans-Golgi. The potential glycoforms include the high mannose, hybrid, and complex structure. Reprinted from Li et al., 2017 [5] with permission of the copyright owner.

Glycosylation is the most common post-translational modification of proteins. It is a complex process that results in a great diversity of carbohydrate–protein bonds and glycan structures. It is known that it has a great impact on protein structures and functions [8]. Glycosylation of IgG is an enzyme-directed chemical reaction that occurs in the endoplasmic reticulum (ER) and the Golgi apparatus of the cell. Initially, a Glc3Man9GlcNAc2 oligosaccharide is transferred to Asn-297 of the IgG heavy chain via an oligosaccharyltransferase complex in the ER. Subsequently, the *N*-glycans are subjected to a sequence of consecutive modifications by sets of glycosidases and glycosyltransferases [9]. Polypeptide-associated Glc3Man9GlcNAc2 is trimmed by glucosidases I and II and endo-mannosidase in the lumen of the ER, resulting in the removal of three Glc residues and a mannose residue to produce Man8GlcNAc2 (Figure 3) [5]. In the cis-Golgi, the Man8GlcNAc2 is sequentially subjected to two class I α-mannosidases that act particularly on α-1,2-Man residues to produce the core Man5GlcNAc2 glycan for additional modification in the medial and trans-Golgi, mediated by GlcNAc transferases I, II, and III (GnT I, II, and III), α-1,6-fucosyltransferase (FUT8), galactosyltransferases (GalT), and sialyltransferases (SiaT) [3,5,9].

## 3. *N*-Glycosylation Impact on mAb Structure and Effector Function

The amount and nature of glycosylation can dramatically affect the behavior of endogenous and recombinant IgGs. The most commonly described roles for glycosylation are related to receptor binding and Fc effector functions. However, the glycosylation profile of an IgG can also substantially affect its PK and distribution. In order to understand the possible manipulations and reasons behind glycosylation and glycoengineering, the reader is also directed to references [3,4,5,6,7,10] for a thorough overview describing the current understanding of glycosylation pattern (and normal variation), normal PK, and effector functions in IgG. As such, Fc glycosylation has great influence on mAbs’ efficacy, stability, safety, immunogenicity, PK, and PD.

### 3.1. Impact of Fc Glycosylation on Structure

**Figure 4:**
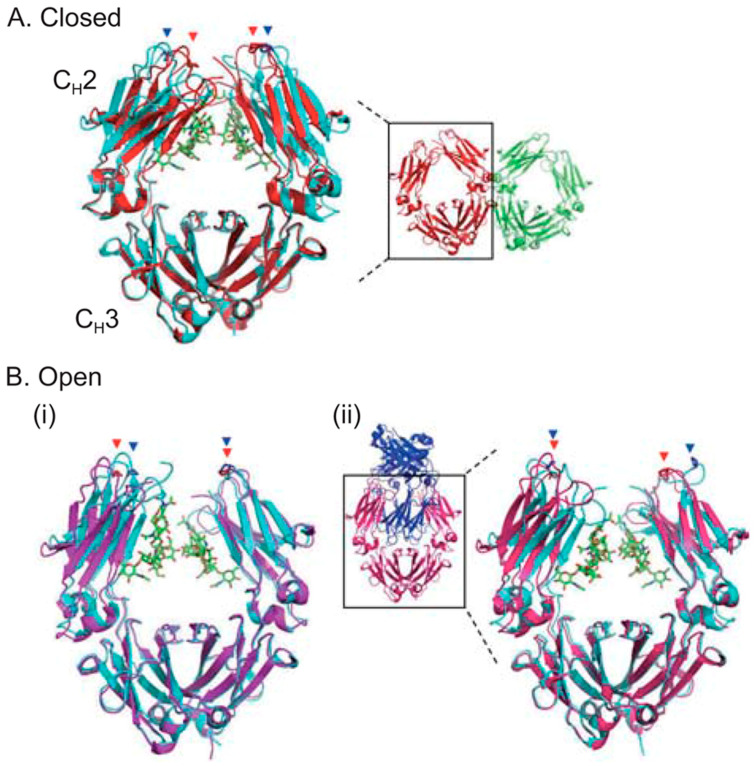
Comparison of non-glycosylated and glycosylated Fc structures. (**A**) Closed conformation of the non-glycosylated Fc. Overall structure of the two aglycosylated Fc molecules is shown in red and green, and the Fc shown in red is superimposed with the glycosylated Fc. (**B**) Open conformation of the non-glycosylated Fc. Overall structure of the two interlocked Fc molecules is shown in pink and blue. The Fc shown in pink is superimposed with the glycosylated Fc. The Fc glycans are shown in green sticks. The Pro329 residues located in the FG loop of the CH2 domains are indicated by red and blue arrowheads for the non-glycosylated and glycosylated CH2 domains, respectively. Reproduced from Mimura et al., 2018 [6] with permission of the copyright owner.

It is well established that the glycan structures can directly affect IgG through altering the conformation of the Fc domain [11]. *N*-glycans have essential structural supportive functions. They play a critical role in the stability of CH2 domain of IgGs, which binds to the glycans via extensive non-covalent interactions that reduce the dynamics of CH2 and aid in CH2 folding. Deglycosylation makes mAbs thermally less stable and more prone to unfolding and degradation [10]. Furthermore, removal of sugar residues leads to the generation of a “closed” conformation while the fully galactosylated IgG-Fc correlates with “open” conformation, which may be most favorable for FcγR binding [12] (Figure 4) [6].

### 3.2. Impact of Fc Glycosylation on Immunogenicity

As mentioned above, glycosylated mAbs can alter their safety and immunogenicity. Glycan patterns are highly variable since they depend on the host glycosylation machinery. Thus, different host cells can produce different recombinant antibodies with different glycoforms. Most therapeutic recombinant antibodies are Chinese hamster ovary (CHO)-derived recombinant IgG molecules, and some are made in murine myeloma cell lines NS0 and SP2/0. Recombinant antibodies produced in CHO cells are glycosylated similarly to natural human IgG. On the other hand, recombinant human IgGs derived in murine myeloma cells can have different glycoforms because they add sugars which are not normally found in the human IgG [3,5]. Terminal-sugar residues expressed in non-human glycoforms that are not normally found in endogenous serum IgGs could be highly immunogenic in humans [13]. Immunogenicity of these therapeutic antibodies can lead to reduced efficacy and safety and cause anti-drug Ab responses (ADA) and hypersensitivity reactions. Therefore, the expression system (bacteria, yeast, insect, plant, or mammalian cells) that is used to generate recombinant mAbs is crucial and has tremendous influence on the mAb function in vivo [14].

Glycoproteins that are produced in yeasts, plants, and insect cells usually have high-mannose contents, which can increase immunogenicity of recombinant mAbs [15]. Lam et al. have demonstrated that antigen mannosylation significantly increases protein immunogenicity in mice [16]. Most therapeutic mAbs, however, have very low levels of high-mannose content [17]. Moreover, terminal sialic acids of therapeutic mAbs derived in non-human cells, such as murine myeloma cell lines, have been shown to be a possible factor that cause immunogenicity in patients since they express the N-glycolylneuraminic acid (NGNA) form of sialic acids that are not normally found in human IgGs [18]. The main reason behind this significant immunogenicity could be NGNA-specific antibodies that have been found to be expressed by all humans [19]. More specific investigations by Qian and coworkers have reported that Cetuximab, a murine myeloma cell-derived novel therapeutic monoclonal antibody that contains NGNA, caused immune interaction with NGNA-specific antibodies [20]. Because of these findings, assessment of the immunogenicity of therapeutic Abs is a critical quality attribute that should be considered with respect to the manufacturing of these therapeutic glycoproteins.

### 3.3. Impact of Fc Glycosylation on Pharmacokinetics

Clearance has a critical impact on the efficacy of therapeutic antibodies. Monoclonal antibodies are high-molecular weight drugs that are large complex proteins (approximately 150 kDa) that are not eliminated through kidney filtration. In addition, they can escape fast degradation in the lysosomes through the neonatal Fc receptor (FcRn) recycling mechanism [21]. The binding of Fc to the neonatal Fc receptor at the CH2–CH3 domain plays a critical role in the PK properties of IgG molecules. Recycling of antibodies results in long half-life of IgGs in the serum (up to 4 weeks) [22]. Roopenian et al. conducted experiments on FcRn knockout mice and they have concluded that FcRn is responsible for protecting IgG from catabolism [22]. Both glycosylated and deglycosylated IgGs bind equally to the (FcRn) receptor [23]. Therefore, the interaction between FcRn and IgG is independent of the Fc glycans due to their protected and buried position within the antibody structure. The significance of Fc glycosylation in the PK of therapeutic mAbs can be examined by comparing the biological activities of glycosylated IgG with either enzymatically deglycosylated IgGs or by preparing aglycosylated IgGs (bearing Asn-297 mutation) using molecular biology techniques. Several studies have compared the biological activity and PK properties of antibodies with different glycoforms in humans and animals [24,25].

Liu et al. confirmed that glycosylation is not required for an IgG antibody’s long half-life after they characterized aglycosylated IgGs by chemical modification and genetic engineering [23]. These animal studies demonstrated that the PK profile of an aglycosylated IgG1 mAb with an Asn-297 mutation was almost identical to that of the glycosylated form. Another clinical trial conducted in 2009 by Clarke et al. also demonstrated that aglycosylated mAb ALD518 (clazakizumab), a humanized anti-human IL-6 IgG1 produced in yeast, had a normal PK in humans and animals. In their phase I clinical trial, the circulating half-life for ALD518 was 20–32 days, which is consistent with the half-life of a normal human IgG1 [26]. Moreover, Abuqayyas and colleagues found that 8C2, a mouse IgG mAb, exhibited similar PK and tissue distribution in both FcγR knockout mice and in wild type mice [27]. Similar PK properties of glycosylated and non-glycosylated IgGs confirm that antibody clearance in humans and animals is not significantly affected by Fc glycan removal [10,24,28].

### 3.4. Effect of Terminal Mannose on Pharmacokinetics

**Figure 5:**
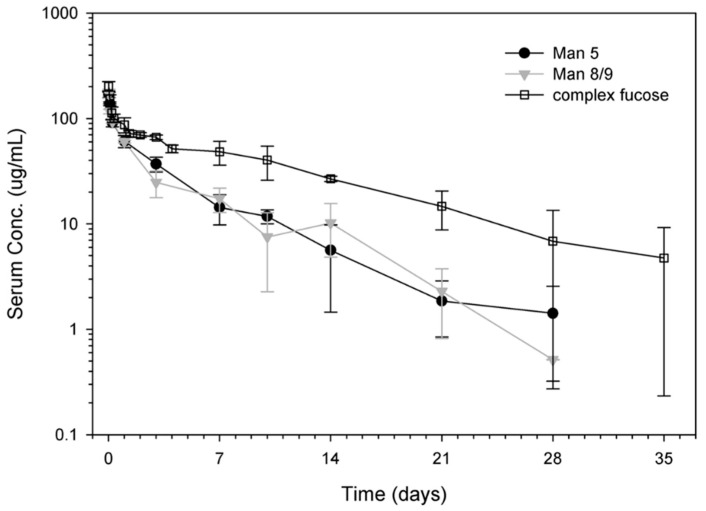
The influence of high-mannose glycans on the pharmacokinetics (PKs) of mAbs. Pharmacokinetic profiles of mAb variants in athymic nude mice. The two groups conducted in this study were mAb with >99% Man5 (solid black circle) and mAb with >99% Man8/9 (solid gray triangle). The complex-fucosylated profile (open squares) was from a separate study conducted in a similar fashion to the current study. Reproduced from Yu et al., 2012 [25] with permission of the copyright owner.

Circulating glycoproteins can be cleared from the blood by receptors that recognize specific glycan forms. Glycan receptors that are involved in the clearance of glycoproteins include the mannose receptor (ManR) and the asialoglycoprotein receptor (ASGPR). The asialoglycoprotein receptors bind to terminal Gal residues and the ManR bind to glycoproteins with terminal Man or GlcNac sugars. Glycan binding to these receptors expressed on tissues was considered to have potential effects on the PK of antibodies bearing these terminal sugars and to cause faster removal from circulation [25]. Consistent with this, Kanda et al. demonstrated that IgG antibodies with high-mannose glycoforms have shorter half-life compared to those with the complex-type glycans in mice [29]. Yu et al. conducted a PK study in mice, and they determined the clearance rate of antibodies bearing Man8/9 and Man5 glycan. They showed that the antibodies bearing the high mannose glycoform were cleared faster compared with antibodies bearing the fucosylated complex glycoform, while the PK properties of antibodies with Man8/9 and Man5 glycoforms appeared similar (Figure 5) [25]. In agreement with previous human studies, Goetze and coworkers observed faster elimination of therapeutic IgGs containing Fc high-mannose glycans from circulation compared to other glycoforms [17]. In addition, differences in high-mannose structural isoform clearance rates in humans were reported by Chen et al., but these investigators suggested that changes in the serum half-life of mAbs bearing high mannose glycoforms were actually due to glycan cleavage [24]. Another investigation done by Millward et al. reached contradictory conclusions. They found no significant difference in serum half-life in mice between high-mannose IgG type and complex IgGs [30]. In summary, high-terminal mannose content appears to be an important point that should be considered as it may affect PK properties and efficacy of therapeutic antibodies. Because of the above findings, most mAbs for clinical use possess relatively low-terminal high-mannose glycan content.

In general, glycans that have a major impact on PK of mAbs include mannose, sialic acids, galactose, and fucose [3,25] (Figure 5). The negatively-charged sialic acids attached to the terminus of glycan chains have been shown to affect half-life for many glycoproteins. It was found that IgGs with exposed terminal Gal (after removal of sialic acid) resulted in a decreased half-life in mice and localization in the liver [3]. To date, the PK properties of different glycan compositions in approved antibody-based therapeutics have not yet been investigated in the clinic.

## 4. Impact of Fc Glycosylation on Pharmacodynamics

The oligosaccharides of the IgG-Fc play a critical role in activation of FcγRs and complement C1. FcγR-mediated effector functions result in the killing of the target cell. FcγRs are responsible for ADCC effector function and, while the receptor C1q mediates CDC. Many studies have found that the lack of glycosylation noticeably decreases the binding affinity to FcγRI and eliminates the binding to FcγRII and FcγRIII receptors [31,32].

### 4.1. Sialic Acid

Sialic acids are present in human serum IgGs as *N*-acetylneuraminic acid (NANA) attached to a terminal galactose by an α-2,3 or α-2,6 linkage. Recombinant monoclonal antibodies expressed in CHO cell line also have NANA, but it is only attached by α-2,3 linkage [18]. On the other hand, monoclonal antibodies produced in NS0 and SP2/0 cell lines have NGNA, a sialic acid form produced by hydroxylation of NANA utilizing cytidine monophosphate *N*-acetylneuraminic acid hydroxylase enzyme which is absent in human and CHO cells under normal conditions [33]. Typically, the level of sialic acid in human endogenous IgGs is ~11%–15% [18,34]. Studies to date that explore the effects of sialic acid on Fcγ receptors binding are inconclusive. Scallon and coworkers studied pairs of monoclonal human IgG Abs produced in mouse hybridoma cell lines with different amounts of sialic acid in their Fc glycans [35]. They demonstrated that a higher content of terminal sialylation was correlated with decreased activity in ADCC and lower-affinity binding to FcγRIIIa on natural killer (NK) cells in vitro. Similarly, Kaneko et al. reported that Fc sialylation affects antibody effector functions including reduction of ADCC in both in vitro and in vivo [36].

However, another in vitro study investigated the influence of sialic acid on IgG1 effector functions using different glycosylated forms of a single drug with various levels of sialylation generated by in vitro glycoengineering [37]. They found that terminal sialylation had no impact, neither positive nor negative, on ADCC activity, FcγRI, and RIIIa receptors, but slightly improved affinity to FcγRIIa was reported [37]. Furthermore, full sialylation of human monoclonal IgG1 was reported to interfere with the induction of CDC in vitro [38].

Recently, Fc sialylation has drawn scientists’ attention as it has been attributed to increased anti-inflammatory responses to intravenous Ig (IVIG) for the treatment of autoimmune and inflammatory diseases [36,39]. IVIG suppresses inflammation by binding to inhibitory FcγRIIb. Sialylated IgG initiates anti-inflammatory effects by binding to the murine C-type lectin-like receptor-specific intracellular adhesion molecule-grabbing non-integrin R1 (SIGN-RI) (DCSIGN in humans) expressed by macrophage and dendritic cells. As a result, FcγRIIb expression will be upregulated and Treg cell populations will expand, leading to significant suppression of inflammatory responses [40,41]. Kaneko et al. approved that sialylated human IgG has elevated anti-inflammatory activity compared to the desialylated IgG utilizing a mouse model of rheumatoid arthritis [36]. However, these findings in mice were contradicted by a study of rheumatoid arthritis during pregnancy [42]. This study showed that remission of rheumatoid arthritis was associated with galactosylation independently of sialylation. In summary, sialylated glycans collectively have both positive and negative influences on IgG effector functions, making it crucial to quantitate the sialylation of mAbs headed for the clinic, especially to treat autoimmune conditions. To date, the functions of different Fc-sialylated glycans in approved antibody-based therapeutics have not yet been investigated in the clinic.

### 4.2. Terminal Galactose

**Figure 6:**
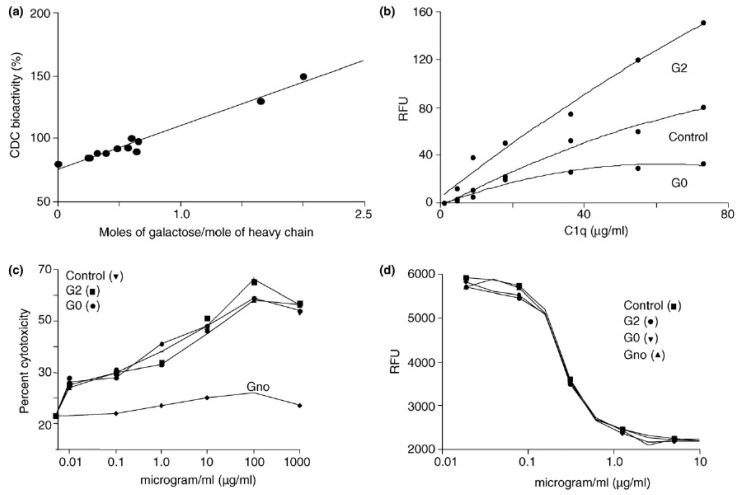
Increase in terminal Gal content increases complement-dependent cytotoxicity (CDC) activity (**a**) and C1q binding (**b**) of rIgGs, but does not affect antibody-dependent cell-mediated cytotoxicity (ADCC) activity (**c**) and antigen binding expressed as relative fluorescence units (RFU)(**d**). G2, G0, and/or Gno (no glycans) glycoforms of Rituxan and/or Herceptin were prepared by in vitro glycosylation methods. These glycoforms, along with control antibody samples (untreated), were subjected to CDC (Rituxan glycoforms), C1q binding (Rituxan glycoforms), ADCC (Herceptin glycoforms), and antigen binding to HER2-ECD (Herceptin glycoforms). Rituxan is a chimeric antibody against CD20 and elicits CDC activity but shows very little ADCC activity. Herceptin is a humanized antibody against HER2-neu antigen and elicits ADCC activity but no CDC activity. Reproduced from Raju 2008 [8] with permission of the copyright owner.

Recombinant mAbs and the human endogenous IgG Fc region have biantennary complex oligosaccharides with either zero, one, or two terminal galactose moieties, which are the three major glycoforms (G0, G1, or G2) [18,34,43]. The impact of terminal galactose residue on IgG biological functions has been investigated in many studies. Whereas the terminal Gal residue content has shown to play an important role in CDC activity of IgG, the ADCC activity does not seem to be affected by galactosylation of an IgG mAb. Hodoniczky and colleagues have remodeled the Fc *N*-glycans of recombinant therapeutic monoclonal antibody products, Rituxan and Herceptin, in vitro, yielding degalactosylated mAb and other products varying in content of GlcNAc [44]. By degalactosylation of Rituxan and generating mAbs with various Gal content, they have demonstrated that CDC activities and antibody binding to C1q increase as Gal content increases [8,44] (Figure 6). Lower affinity to C1q is due to hydrophobic and hydrophilic interactions between terminal Gal residue and protein, which alter the conformation of the CH2 domain [12]. They confirmed that ADCC activity is not influenced by terminal Gal residue content [44]. Nevertheless, despite some of these contradictory results, galactosylation can induce a positive impact on the binding affinity of the IgG1 to FcγRIIa and FcγRIIIa receptors and ADCC activity [37]. A recent in vitro study also showed that Fc-galactosylation of rituximab enhances CDC activities compared to the degalactosylated glycoform and improvement of C1q binding eventually leads to tumor cell lysis [45]. However, these findings apply to IgG1, but not other subclasses of mAbs. Thus, further detailed, specific investigations of the effects of galactosylation on other IgG subclasses effector functions such as ADCC and CDC are needed.

### 4.3. Bisecting N-Acetylglucosamine

**Figure 7:**
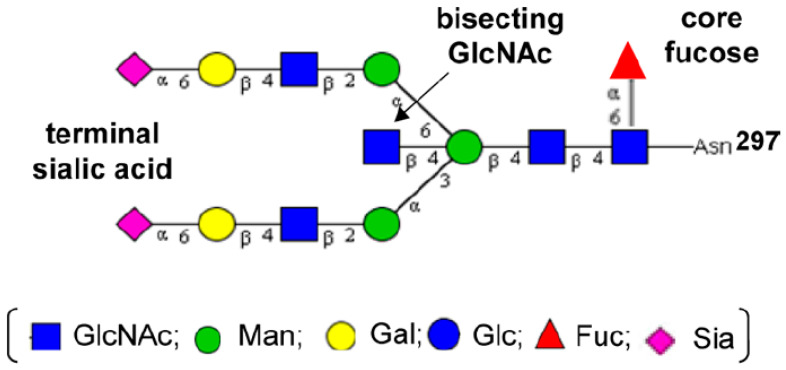
The structure of a full-length biantennary *N*-glycan attached to the Asn-297 in the Fc domain and containing bisecting GlcNAc residue. Reproduced from Huang et al., 2012 [50] with permission of the copyright owner.

Approximately 10% of human serum endogenous IgGs glycoforms have bisecting GlcNAc residues. Recombinant antibodies generated in CHO cells do not contain bisecting GlcNAc because of the lack of active *N*-acetylglucosaminyltransferase-III (GnT-III) needed for synthesis of bisecting GlcNAc containing *N*-glycans [8,32,46] (Figure 7). Addition of a bisecting GlcNAc has been reported to enhance the binding affinity to FcγRIIIa, which causes 10–30-fold higher ADCC activities [47,48]. Although Hodoniczky et al. [44] approved that bisecting GlcNAc enhances ADCC activity by approximately 10-fold independently of the lack of core fucosylation of rituximab remodeled in vitro, a study done by Shinkawa et al. [48] has debated these findings. Since loss of core fucosylation is always associated with in vivo addition of bisecting GlcNAc, Shinkawa and colleagues proposed that the presence of bisecting GlcNAc may not be the main cause of an ADCC activity increase. As such, Shinkawa’s studies demonstrated that the removal of core fucose rather than bisecting GlcNAc has the biggest impact on ADCC activity of therapeutic antibodies [48]. Similar to Shinkawa’s results, Ferrara et al. [49] have reported that antibodies enriched in bisected oligosaccharides have increased ADCC.

### 4.4. Fucose

The core fucose residues are added to the core GlcNAc residue which is linked to the protein via α-1,6 linkage. This complex glycoform is dominantly found in both serum IgG (>80%) and recombinant IgG’s produced in CHO cells (>90%) [32]. Although absence of core fucose residues in Fc glycans has been shown to dramatically improve antibody binding to FcRIIIa and ADCC activity, many studies have demonstrated that the fucosylation level has slight consequences on binding of antibodies to FcγR1, FcγRII, and C1q [48,51,52]. Shields and coworkers used afucosylated anti-HER2 to demonstrate the significant role played by the absence of core fucose in the enhancement of ADCC activity of IgG [51]. About 100-fold greater ADCC exhibited by afucosylated anti-HER2 compared to fucosylated recombinant IgG has been reported in this study. Furthermore, it has been observed that the binding to FcγRI, C1q, or FcRn was not altered [51]. Using marketed nonfucosylated anti-CD20 IgG1 rituximab, Lida et al. confirmed that nonfucosylated IgG1 mediates very high ADCC at low doses in humans, which enhances the therapeutic potential of the modified mAb [53]. Another study has shown that higher binding affinity of afucosylated IgG to Fcγ RIIIa apply for all IgG subclasses [54]. Inclusively, afucosylation of mAb leads the greatest influence on ADCC enhancement, which mediates the efficacy of potential therapeutic recombinant antibodies. Therefore, many recombinant IgGs modified via glycoengineering strategies to generate low-fucose antibodies are currently under investigation in human clinical trials to improve the clinical efficacy of these therapeutics. A classic example of producing low-fucose content antibodies is the anti-CD20 antibody obinutuzumab, which was approved by the US Food and Drug Administration (FDA) in 2013 for the treatment of non-Hodgkin’s lymphoma and chronic lymphocytic leukemia. Obinutuzumab showed significantly increased ADCC activity compared with the prototype antibody (rituximab). The same engineered cell line was used to produce a nonfucosylated anti-CD20 antibody (mogamulizumab) that showed a 100-fold increase in ADCC activity compared with the nonglycoengineered rituximab. As such, mogamulizumab was approved for the treatment of adult T cell leukemia/lymphoma in Japan.

### 4.5. High Mannose

Typically, high mannose content in Fc glycan of IgG varies from five to nine mannose molecules linked to the core GlcNAc. Although about 0.1% of human serum endogenous IgG’s contain Fc glycan with high mannose (mostly Man5GlcNAc2 structure), high mannose glycoforms content varies with cell lines and can represent up to 10% of recombinant IgG [20]. Different studies exploring high mannose type Fc glycans have shown that mannose content can impact antibody effector functions. Zhou and his team have demonstrated that the presence of high mannose structures result in enhanced ADCC activity and increased IgG binding affinity for the FcγRIIIa receptor [55]. However, it is unclear whether enhancement in ADCC activity is due to the absence of fucose, since high mannose type glycans possess no core fucose residues [20]. It has also been shown that the IgG with high mannose structures have a negative impact on CDC activity because of lower binding affinity for C1q [55]. Consistently, Kanda et al. have reported similar results that high mannose structures can lead to reduced C1q binding and complement activation [29]. Nevertheless, it was shown that mAbs with high mannose glycan exhibit higher ADCC in the same study. In conclusion, high mannose type Fc glycans have a positive effect on ADCC activity but a negative impact on CDC activity of IgG molecules.

## 5. Glycoengineering

Since different glycoforms have positive or negative effects on antibody effector functions, it is necessary to develop Fc glycoengineering strategies to facilitate the generation of therapeutic mAbs with consistent and homogenous glycoforms to improve their therapeutic efficacy. Although much progress in cell glycoengineering has already been achieved and important improvements on glycan quality have been accomplished, it is still very challenging to produce IgGs with highly homogenous glycoforms in host cells. The current Fc glycoengineering strategies include host cell glycoengineering and in vitro chemoenzymatic glycosylation remodeling.

### 5.1. Cell Glycoengineering

Antibody glycosylation is the result of a multistep process. Host cell-based glycoengineering alters glycoforms by genetically modifying important mediators in the glycan biosynthetic pathways to enhance production of desired glycoforms [56]. This technology has been used recently to generate mAbs with optimized quality and efficacy, and focusing on Fc defucosylation which produces a significant increase in ADCC activity results due to the absence of core fucose [57]. Various approaches have been used to modify host cells in order to enhance the desired or limit the unwanted glycoforms. One approach selects host cell type, environmental factors, and cell culture conditions. Host cells that have low FUT8 activity, such as rat hybridoma cell line YB2/0, allow production of recombinant glycoproteins with low core fucose [58,59]. Recombinant mAbs derived from CHO cells exhibit low sialic acid levels because of the absence of α-2,6-sialyltransferase in these cells [60]. Therefore, this cell type is an attractive alternative for the production of mAbs with low sialic acid content. Moreover, cell culture conditions can be modified to favor antibody glycoforms homogeneity. Crowell et al. have reported that feeding the culture with uridine, manganese chloride, and galactose could result in higher CDC activity of mAb due to increased terminal galactose [61]. Another study used 2-fluorofucose, a fucose analogue, to inhibit fucosylation in vitro and produce fucose-deficient antibodies [62].

Another approach in host cells glycoengeneering uses inhibitors of the enzymes that synthesize *N*-linked oligosaccharide chains to alter host biosynthesis pathways. Enzyme inhibitors prevent the addition of outer arm sugar residues including fucose [63]. For example, the addition of ER α-mannosidase inhibitors, deoxymannojirimycin and kifunensine, results in the generation of high mannose (Man9GlcNAc2) glycoform. Another example is that ER glucosidases I and II inhibitors include deoxynojirimycin and castanospermine which arrest mAb in Glc3Man9GlcNAc2 glycoform [63].

A third approach is genetic modulation of the host glycan biosynthesis pathway. This strategy can be performed by upregulating or downregulating substrate expression. Sullivan’s group succeeded in the generation of defucosylated antibodies by silencing the *GMD* gene responsible for the expression of GDP fucose, the fucose donor [64]. Furthermore, gene editing techniques, such as ZFNs, TALENs, and CRISPR-Cas9, have been widely used to modify *N*-glycosylation pathways. Chan et al. used these techniques to inactivate the GDP-fucose transporter (SLC35C1) in Chinese hamster ovary (CHO) cells. They concluded that inactivating the *Slc35c1* gene results in production of fucose-free antibodies in CHO cells [65]. Alternatively, small interfering RNis (siRNAs) have been used to knock out multiple genes involved in fucosylation. Finally, inactivation of FUT8 and GDP-mannose 4,6-dehydratase *(GMD)* in CHO cells has led to the production of completely afucosylated IgG with enhanced ADCC [66]. For example, to improve ADCC, a significant improvement through cell-based glycoengineering has been previously reported with the first approved mAbs mogamulizumab and obinutuzumab. Mogamulizumab (POTELIGEO^®^, KW0761) is a humanized mAb which uses a FUT8 knockout CHO cell line to produce mAbs with nonfucosylated glycan mixtures [66]. Obinutuzumab (Gazyva™, GA-101) is derived from Roche GlycoMAb^®^ technology which overexpresses GnTIII [46,47]. Once the GnT-III adds a bisecting GlcNAc to an oligosaccharide, the core-fucosylation is inhibited. Both technologies produce therapeutic mAbs with enhanced ADCC activity.

### 5.2. Chemoenzymatic Glycoengineering

**Figure 8:**
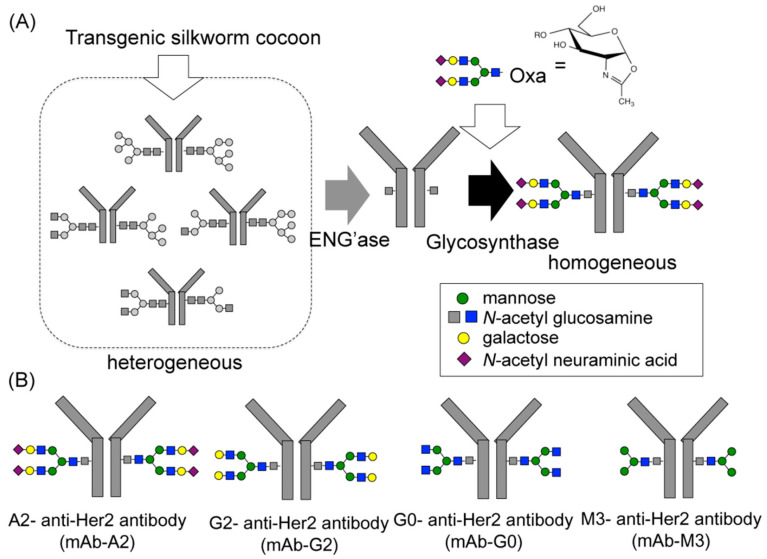
(**A**) Schematic representation of chemoenzymatic synthesis using ENG’ase and glycosynthase. (**B**) Diagram of the homogeneous glycosylated mAb with M3 (mAb-M3), G0 (mAb-G0), G2 (mAb-G2), and A2 (mAb-A2). Reproduced from Kurogochi et al., 2015 [68] with permission of the copyright owner.

Although much successful work in cell glycoengineering has been done to generate therapeutic mAbs with specific glycoforms, it is still very difficult to produce optimized IgGs with homogeneous glycoforms. To accomplish this, chemoenzymatic glycosylation of IgG antibodies provides a new avenue to remodel Fc *N*-glycan from a heterogeneous *N*-glycosylation pattern to a homogeneous one. The Protocol of chemoenzymatic synthesis includes deglycosylation of IgG antibodies using ENG’ase (endo-β-*N*-acetylglucosaminidase) leaving the innermost GlcNAc with or without core fucose at the *N*-glycosylation site. After preparation of glycan oxazolines as donor substrates, a transglycosylation step is used with ENGase-based glycosynthase [66,67,68] (Figure 8A), and then prepared the glycoengineered mAbs with homogenous *N*-glycans (M3, G0, G2, and A2) via enzymatic reaction (Figure 8B).

**Figure 9:**
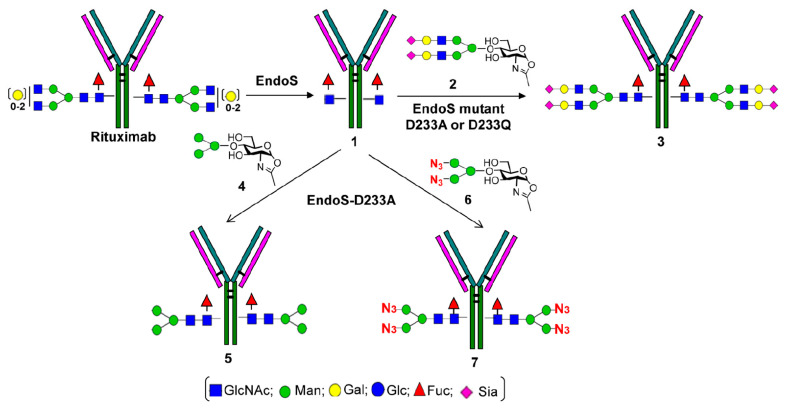
Chemoenzymatic remodeling of rituximab to prepare homogeneous and selectively modified glycoforms. Reproduced from Huang et al., 2012 [50] with permission of the copyright owner.

There are various ENGases mutants (EndoS D233Q, EndoA N171A, EndoA E173Q, EndoMN175A, and EndoM N175Q) that exhibit transglycosylation activity, which have been engineered to have different substrate specificities and limitations [50,69]. As an example, Huang and coworkers [50] generated two glycosynthase mutants (EndoS-D233A and D233Q) to transform rituximab from mixtures of G0F, G1F, and G2F glycoforms to well-defined homogeneous glycoforms. Using EndoS glycosynthase mutants permitted the production of a fully sialylated (S2G2F) glycoform that shows enhanced anti-inflammatory activity of IVIG’s Fc glycans, and a nonfucosylated G2 glycoform that favors increased FcγIIIa receptor-bindings and ADCC activity of mAbs [50] (Figure 9).

While many investigations have demonstrated that Endo-S is limited to action on the complex-type, a more recent study described Endo-S2 glycosynthases (D184M and D184Q) that have relaxed substrate specificity and act on transferring three major types (complex, high-mannose, and hybrid type) of *N*-glycans [70]. Collectively, chemoenzymatic glycoengineering technology may be used to develop therapeutic monoclonal antibodies that have homogenous glycoforms, which may circumvent all current efficacy and function quality issues.

### 5.3. Glycoengineering for Site-Specific Antibody-Drug Conjugation

Antibody-drug conjugates or ADCs are emerging as powerful reagents for the selective delivery of highly toxic drugs to target cells. These relatively novel agents combine the ability of mAbs to bind antigen positive tumor cells with the highly potent killing activity of a cytotoxic drug. In one of the several approaches to obtain structurally-defined, homogeneous antibody–drug conjugates, the Fc glycans of the antibody is engineered for site-specific conjugation [71]. As discussed above, IgGs carry a highly conserved *N*-glycan at the Asn-297 of the Fc domain. Several terminal residues of glycoproteins, including fucose, galactose, and sialic acids that contain vicinal cis diols, can be oxidized selectively with mild periodate (NaIO4) treatment to generate aldehyde groups, which can be further functionalized with other groups, including hydrazides and aminooxy groups for chemoselective conjugation. However, antibody glycosylation is highly heterogeneous, and contains a mixture of galactose and core fucose. As a result, direct oxidation recombinant antibodies usually led to heterogeneous mixtures of the conjugates. For example, to have better control of the homogeneity of ADCs, researchers developed a CHO cell line that could control Fc *N*-glycosylation at the G0F glycoform, where fucose could be selectively oxidized. Thus, treatment of the G0F antibody with mild NaIO4 selectively oxidized the fucose moiety to provide an aldehyde derivative. In contrast to core fucose, oxidation of sialic acid can take place under relatively different conditions because their cis diols are less hindered and more susceptible to periodate oxidation. The advantage of this site-selective modification at the conserved *N*-glycan does not change the IgG structure and thus usually will not affect the antibody’s inherent affinity for its antigen. A thorough overview on recent approaches behind glycoengineering of antibodies for site-specific antibody–drug conjugation is described in reference [71].

Today, there are 4 antibody-drug conjugates approved by the US FDA, including Genentech/Roche’s Kadcyla^®^ (HER2-specific trastuzumab-drug conjugate) used for the treatment of metastatic breast cancer, Seattle Genetics’s Adcetris^®^ (CD30-specific brentuximab-drug conjugate) used for treatment of relapsed Hodgkin’s lymphoma, Pfizer/Wyeth’s Besponsa^TM^ (CD22-specific inotuzumab-drug conjugate) used for relapsed or refractory B cell precursor acute lymphoblastic leukemia, and more recently, Wyeth Pharmaceuticals’ Mylotarg^TM^ (CD33-specific gemtuzumab-ozogamicin conjugate) used for the therapy of acute myelogenous leukemia.

## 6. Conclusions

In summary, therapeutic mAbs are large, complex, and heterogeneous glycoproteins. They are typically glycosylated at amino acid position 297 in the Fc region. The *N*-glycosylation is crucial for antibody structure and effector functions. The presence or absence of different terminal sugars of Fc glycans can have a significant impact on the PK, PD, and immunogenicity of mAbs (Table 1). Although several studies investigated the correlation between PD and *N*-glycosylation, the results were often contradictory. Whereas high mannose content was shown to significantly impact PK by decreasing antibody half-life, the impact of other glycans on PK is still not fully understood [72]. Collectively, glycosylation is not essential for IgGs’ long half-life, and FcRn is the main factor that maintains IgGs’ circulation time. Furthermore, therapeutic IgGs derived from non-human cells can be immunogenic as they may express terminal sugar residues that are not naturally found in human serum IgGs, such as sialic acid NGNA. This immunogenicity can decrease the drug efficacy and cause hypersensitivity reactions. For the effects of glycoform patterns on PD and IgG effector functions, the presence of core fucose can interfere with FcγRIIIa binding and ADCC activity of therapeutic antibody. Therefore, the removal of fucose should be considered to enhance ADCC activity of monoclonal antibody drugs. On the other hand, galactosylation can improve the efficacy and quality of mAbs by increasing antibody binding to C1q and CDC of mAbs. Although progress has occurred, there is still much important work to address the unsolved underlying mechanisms that regulate the relationship between changes in Fc-glycan structures and the efficacy and quality of therapeutic monoclonal antibody functions. Due to the critical role of glycosylation and the great impact of different glycans on therapeutic monoclonal antibodies, the need for developing novel glycoengineering strategies has emerged in the last decade [5]. These strategies offer a new route to produce homogenous IgGs with desired glycoforms in order to enhance efficacy and functionality of therapeutic glycoproteins. Glycoengineering techniques which include glycoengineering of cell lines and chemoenzymatic glycoengineering approaches [50,68] are evolving and offer promising novel avenues to develop stable and safer mAbs, which is ultimately linked to lower risk of immunogenicity and higher therapeutic efficacy in humans [5,73,74,75,76]. As such, understanding the ways to control the Fc-glycan heterogeneity is essential to the successful clinical development of antibody-based drugs, which can be used to predict their PK/PD during early clinical development and to ensure faster results. This information can appropriately inform manufacturing process development so that these processes are more finely adjusted to deliver the desired Fc glycosylation [77].

In respect to biosimilar development, site-specific glycosylation is also considered crucial in correlating distinct product attributes with observed in vivo effects [13,78]. In this context, an in-depth method for the characterization and analysis of manufactured biosimilar products is required for the production of optimal and consistent biosimilar therapeutic products. A better understanding of the relationship between glycosylation patterns and clinical performance is also of major importance for biosimilar development, and can be used to develop safe and efficacious antibody-based products on the market. In conclusion, antibody glycosylation is necessary to optimize the stability, safety, functionality, and efficacy of therapeutic IgG antibodies. Furthermore, new methods are being applied to generate the next generation of therapeutic mAbs for the treatment of a wide spectrum of human diseases.

To date, antibody-based products are still presenting academia and the biotechnology industry with novel challenges in terms of glycan characterization, stability, and in vivo behavior. Although many studies have been conducted evaluating the various effects of glycosylation on their physicochemical properties and patterns including their importance to biosimilarity [72], our understanding of how glycosylation translates to potential pharmacologic effects and toxicities is still incomplete. Additional investigations are therefore warranted to obtain a clearer pictu re of its importance to antibodies as a vital and important class of drugs.

## Figures and Tables

**Table 1:** Summary of potential effects of the most prevalent Fc-glycans on the pharmacokinetics (PK) and pharmacodynamics (PD) of monoclonal antibodies (mAbs). N-Glycolylneuraminic acid form of sialic acid (NGNA).

Fc-Glycans	Potential Effects	References
**Fucose**	Absence of core fucose enhances: FcγRIIIa binding ADCC activity	[48,51,52,53,54]
**Galactose**	Enhances antibody binding to C1q and CDC	[44,45]
**Sialic acid**	Anti-inflammatory activity NGNA reduces FcγRIIIa binding and ADCC activity NGNA may be immunogenic in human Removal of sialic acid decreases half-life	[36,39] [35,36] [18,19,20] [3,25]
**High Mannose**	Decreases half-life Increases FcγRIIIa binding and ADCC activity Decreases antibody binding to C1q and CDC	[17,25,29] [55] [29,55]
**Bisecting GlcNAc**	Increases FcγRIIIa binding and ADCC activity	[44,47,48,49]

## References

[1] Fang J., Richardson J., Du Z., Zhang Z. (2016). Effect of Fc-glycan structure on the conformational stability of IgG revealed by hydrogen/deuterium exchange and limited proteolysis. Biochemistry.

[2] Kohler G., Milstein C. (1975). Continuous cultures of fused cells secreting antibody of predefined specificity. Nature.

[3] Liu L. (2015). Antibody glycosylation and its impact on the pharmacokinetics and pharmacodynamics of monoclonal antibodies and Fc-fusion proteins. J. Pharm. Sci..

[4] Bakhtiar R. (2012). Therapeutic recombinant monoclonal antibodies. J. Chem. Educ..

[5] Li W., Zhu Z., Chen W., Feng Y., Dimitrov D.S. (2017). Crystallizable fragment glycoengineering for therapeutic antibodies development. Front. Immunol..

[6] Mimura Y., Katoh T., Saldova R., O’Flaherty R., Izumi T., Mimura-Kimura Y., Utsunomiya T., Mizukami Y., Yamamoto K., Matsumoto T. (2018). Glycosylation engineering of therapeutic IgG antibodies: Challenges for the safety, functionality and efficacy. Protein Cell.

[7] Cymer F., Beck H., Rohde A., Reusch D. (2018). Therapeutic monoclonal antibody N-glycosylation–structure, function and therapeutic potential. Biologicals.

[8] Raju T.S. (2008). Terminal sugars of Fc glycans influence antibody effector functions of IgGs. Curr. Opin. Immunol..

[9] Butters T.D. (2002). Control in the N-linked glycoprotein biosynthesis pathway. Chem. Biol..

[10] Higel F., Seidl A., Sörgel F., Friess W. (2016). N-glycosylation heterogeneity and the influence on structure, function and pharmacokinetics of monoclonal antibodies and Fc fusion proteins. Eur. J. Pharm. Biopharm..

[11] Borrok M.J., Jung S.T., Kang T.H., Monzingo A.F., Georgiou G. (2012). Revisiting the role of glycosylation in the structure of human IgG Fc. ACS Chem. Biol..

[12] Krapp S., Mimura Y., Jefferis R., Huber R., Sondermann P. (2003). Structural analysis of human IgG-Fc glycoforms reveals a correlation between glycosylation and structural integrity. J. Mol. Biol..

[13] Barbosa M.D. (2011). Immunogenicity of biotherapeutics in the context of developing biosimilars and biobetters. Drug Discov. Today.

[14] Beck A., Wagner-Rousset E., Bussat M.-C., Lokteff M., Klinguer-Hamour C., Haeuw J.-F., Goetsch L., Wurch T., Dorsselaer A., Corvaia N. (2008). Trends in glycosylation, glycoanalysis and glycoengineering of therapeutic antibodies and Fc-fusion proteins. Curr. Pharm. Biotechnol..

[15] Durocher Y., Butler M. (2009). Expression systems for therapeutic glycoprotein production. Curr. Opin. Biotechnol..

[16] Lam J.S., Mansour M.K., Specht C.A., Levitz S.M. (2005). A model vaccine exploiting fungal mannosylation to increase antigen immunogenicity. J. Immunol..

[17] Goetze A.M., Liu Y.D., Zhang Z., Shah B., Lee E., Bondarenko P.V., Flynn G.C. (2011). High-mannose glycans on the Fc region of therapeutic IgG antibodies increase serum clearance in humans. Glycobiology.

[18] Biburger M., Lux A., Nimmerjahn F. (2014). How immunoglobulin G antibodies kill target cells. Adv. Immunol..

[19] Tangvoranuntakul P., Gagneux P., Diaz S., Bardor M., Varki N., Varki A., Muchmore E. (2003). Human uptake and incorporation of an immunogenic nonhuman dietary sialic acid. Proc. Natl. Acad. Sci. USA.

[20] Qian J., Liu T., Yang L., Daus A., Crowley R., Zhou Q. (2007). Structural characterization of N-linked oligosaccharides on monoclonal antibody cetuximab by the combination of orthogonal matrix-assisted laser desorption/ionization hybrid quadrupole–quadrupole time-of-flight tandem mass spectrometry and sequential enzymatic digestion. Anal. Biochem..

[21] Liu L. (2018). Pharmacokinetics of monoclonal antibodies and Fc-fusion proteins. Protein Cell.

[22] Roopenian D.C., Akilesh S. (2007). FcRn: The neonatal Fc receptor comes of age. Nat. Rev. Immunol..

[23] Liu L., Stadheim A., Hamuro L., Pittman T., Wang W., Zha D., Hochman J., Prueksaritanont T. (2011). Pharmacokinetics of IgG1 monoclonal antibodies produced in humanized Pichia pastoris with specific glycoforms: A comparative study with CHO produced materials. Biologicals.

[24] Chen X., Liu Y.D., Flynn G.C. (2008). The effect of Fc glycan forms on human IgG2 antibody clearance in humans. Glycobiology.

[25] Yu M., Brown D., Reed C., Chung S., Lutman J., Stefanich E., Wong A., Stephan J.-P., Bayer R. (2012). Production, characterization and pharmacokinetic properties of antibodies with N-linked Mannose-5 glycans. MABS.

[26] Clarke S., Gebbie C., Sweeney C., Olszewksi N., Smith J. (2009). A phase I, pharmacokinetic (PK) and preliminary efficacy assessment of ALD518, a humanized anti-IL-6 antibody, in patients with advanced cancer (Abstract). J. Clin. Oncol..

[27] Abuqayyas L., Zhang X., Balthasar J.P. (2013). Application of knockout mouse models to investigate the influence of FcγR on the pharmacokinetics and anti-platelet effects of MWReg30, a monoclonal anti-GPIIb antibody. Int. J. Pharm..

[28] Batra J., Rathore A.S. (2016). Glycosylation of monoclonal antibody products: Current status and future. Biotechnol. Prog..

[29] Kanda Y., Yamada T., Mori K., Okazaki A., Inoue M., Kitajima-Miyama K., Kuni-Kamochi R., Nakano R., Yano K., Kakita S. (2006). Comparison of biological activity among nonfucosylated therapeutic IgG1 antibodies with three different N-linked Fc oligosaccharides: The high mannose, hybrid, and complex types. Glycobiology.

[30] Millward T.A., Heitzmann M., Bill K., Längle U., Schumacher P., Forrer K. (2008). Effect of constant and variable domain glycosylation on pharmacokinetics of therapeutic antibodies in mice. Biologicals.

[31] Sazinsky S.L., Ott R.G., Silver N.W., Tidor B., Ravetch J.V., Wittrup K.D. (2008). Aglycosylated immunoglobulin G1 variants productively engage activating Fc receptors. Proc. Natl. Acad. Sci. USA.

[32] Raju T., Briggs J.B., Borge S.M., Jones A.J.S. (2000). Species-specific variation in glycosylation of IgG: Evidence for the species-specific sialylation and branch-specific galactosylation and importance for engineering recombinant glycoprotein therapeutics. Glycobiology.

[33] Ambrogelly A., Gozo S., Katiyar A., Dellatore S., Kune Y., Bhat R., Sun J., Li N., Wang D., Nowak C. (2018). Analytical comparability study of recombinant monoclonal antibody therapeutics. MABS.

[34] Flynn G.C., Chen X., Liu Y.D., Shah B., Zhang Z. (2010). Naturally occurring glycan forms of human immunoglobulins G1 and G2. Mol. Immunol..

[35] Scallon B.J., Tam S.H., McCarthy S.G., Cai A.N., Raju T.S. (2007). Higher levels of sialylated Fc glycans in immunoglobulin G molecules can adversely impact functionality. Mol. Immunol..

[36] Kaneko Y., Nimmerjahn F., Ravetch J.V. (2006). Anti-Inflammatory activity of immunoglobulin G resulting from Fc sialylation. Science.

[37] Thomann M., Schlothauer T., Dashivets T., Malik S., Avenal C., Bulau P., Rüger P., Reusch D. (2015). In vitro glycoengineering of IgG1 and its effect on Fc receptor binding and ADCC activity. PLoS ONE.

[38] Quast I., Keller C.W., Maurer M.A., Giddens J.P., Tackenberg B., Wang L.-X., Münz C., Nimmerjahn F., Dalakas M.C., Lünemann J.D. (2015). Sialylation of IgG Fc domain impairs complement-dependent cytotoxicity. J. Clin. Investig..

[39] Nimmerjahn F., Ravetch J.V. (2008). Anti-Inflammatory actions of intravenous immunoglobulin. Ann. Rev. Immunol..

[40] Anthony R.M., Ravetch J.V. (2010). A novel role for the IgG Fc glycan: The anti-inflammatory activity of sialylated IgG Fcs. J. Clin. Immunol..

[41] Anthony R.M., Nimmerjahn F., Ashline D.J., Reinhold V.N., Paulson J.C., Ravetch J.V. (2008). Recapitulation of IVIG anti-inflammatory activity with a recombinant IgG Fc. Science.

[42] Bondt A., Selman M.H.J., Deelder A.M., Hazes J.M., Willemsen S.P., Wuhrer M., Dolhain R.J.E.M. (2013). Association between galactosylation of immunoglobulin G and improvement of rheumatoid arthritis during pregnancy is independent of sialylation. J. Proteome Res..

[43] Raju T.S., Jordan R.E. (2012). Galactosylation variations in marketed therapeutic antibodies. MABS.

[44] Hodoniczky J., Zheng Y., James D. (2005). Control of recombinant monoclonal antibody effector functions by Fc N-glycan remodeling in vitro. Biotechnol. Prog..

[45] Peschke B., Keller C.W., Weber P., Quast I., Lünemann J.D. (2017). Fc-galactosylation of human immunoglobulin gamma isotypes improves C1q binding and enhances complement-dependent cytotoxicity. Front. Immunol..

[46] Patnaik S.K., Stanley P. (2006). Lectin-resistant CHO glycosylation mutants. Meth. Enzymol..

[47] Davies J., Jiang L., Pan L.-Z., Labarre M.J., Anderson D., Reff M. (2001). Expression of GnTIII in a recombinant anti-CD20 CHO production cell line: Expression of antibodies with altered glycoforms leads to an increase in ADCC through higher affinity for FCγRIII. Biotechnol. Bioeng..

[48] Shinkawa T., Nakamura K., Yamane N., Shoji-Hosaka E., Kanda Y., Sakurada M., Uchida K., Anazawa H., Satoh M., Yamasaki M. (2003). The absence of fucose but not the presence of galactose or bisecting N-acetylglucosamine of human IgG1 complex-type oligosaccharides shows the critical role of enhancing antibody-dependent cellular cytotoxicity. J. Biol. Chem..

[49] Ferrara C., Brünker P., Suter T., Moser S., Püntener U., Umaña P. (2006). Modulation of therapeutic antibody effector functions by glycosylation engineering: Influence of Golgi enzyme localization domain and co-expression of heterologous β1, 4-N-acetylglucosaminyltransferase III and Golgi α-mannosidase II. Biotechnol. Bioeng..

[50] Huang W., Giddens J., Fan S.-Q., Toonstra C., Wang L.-X. (2012). Chemoenzymatic glycoengineering of intact IgG antibodies for gain of functions. J. Am. Chem. Soc..

[51] Shields R.L., Lai J., Keck R., Oconnell L.Y., Hong K., Meng Y.G., Weikert S.H.A., Presta L.G. (2002). Lack of fucose on human IgG1 N-linked oligosaccharide improves binding to human FcγRIII and antibody-dependent cellular toxicity. J. Biol. Chem..

[52] Niwa R., Hatanaka S., Shoji-Hosaka E., Sakurada M., Kobayashi Y., Uehara A., Yokoi H., Nakamura K., Shitara K. (2004). Enhancement of the antibody-dependent cellular. Clin. Cancer Res..

[53] Iida S., Misaka H., Inoue M., Shibata M., Nakano R., Yamane-Ohnuki N., Wakitani M., Yano K., Shitara K., Satoh M. (2006). Nonfucosylated therapeutic IgG1 antibody can evade the inhibitory effect of serum immunoglobulin G on antibody-dependent cellular cytotoxicity through its high binding to Fc RIIIa. Clin. Cancer Res..

[54] Niwa R., Natsume A., Uehara A., Wakitani M., Iida S., Uchida K., Satoh M., Shitara K. (2005). IgG subclass-independent improvement of antibody-dependent cellular cytotoxicity by fucose removal from Asn297-linked oligosaccharides. J. Immunol. Meth..

[55] Zhou Q., Shankara S., Roy A., Qiu H., Estes S., Mcvie-Wylie A., Culm-Merdek K., Park A., Pan C., Edmunds T. (2007). Development of a simple and rapid method for producing non-fucosylated oligomannose containing antibodies with increased effector function. Biotechnol. Bioeng..

[56] Butler M., Spearman M. (2014). The choice of mammalian cell host and possibilities for glycosylation engineering. Curr. Opin. Biotechnol..

[57] Suzuki E., Niwa R., Saji S., Muta M., Hirose M., Lida S., Shiotsu Y., Satoh M., Shitara K., Kondo M. (2007). A nonfucosylated anti-HER2 antibody augments antibody-dependent cellular cytotoxicity in breast cancer patients. Clin. Cancer Res..

[58] Yamane-Ohnuki N., Satoh M. (2009). Production of therapeutic antibodies with controlled fucosylation. MABS.

[59] Urbain R., Teillaud J.L., Prost J.F. (2009). EMABling antibodies: From feto-maternal allo-immunisation prophylaxis to chronic lymphocytic leukaemia therapy. Med. Sci..

[60] Dicker M., Strasser R. (2015). Using glyco-engineering to produce therapeutic proteins. Expert Opin. Biol. Ther..

[61] Crowell C.K., Grampp G.E., Rogers G.N., Miller J., Scheinman R.I. (2006). Amino acid and manganese supplementation modulates the glycosylation state of erythropoietin in a CHO culture system. Biotechnol. Bioeng..

[62] Okeley N.M., Alley S.C., Anderson M.E., Boursalian T.E., Burke P.J., Emmerton K.M., Jeffrey S.C., Klussman K., Law C.-L., Sussman D. (2013). Development of orally active inhibitors of protein and cellular fucosylation. Proc. Natl. Acad. Sci. USA.

[63] Powell L.D. (1995). Inhibition of N-Linked Glycosylation. Curr. Protoc. Mol. Biol..

[64] Sullivan F.X., Kumar R., Kriz R., Stahl M., Xu G.-Y., Rouse J., Chang X.-J., Boodhoo A., Potvin B., Cumming D.A. (1998). Molecular cloning of human GDP-mannose 4,6-dehydratase and reconstitution of GDP-fucose biosynthesis in vitro. J. Biol. Chem..

[65] Chan K.F., Shahreel W., Wan C., Teo G., Hayati N., Tay S.J., Tong W.H., Yang Y., Rudd P.M., Zhang P. (2015). Inactivation of GDP-fucose transporter gene (Slc35c1) in CHO cells by ZFNs, TALENs and CRISPR-Cas9 for production of fucose-free antibodies. Biotechnol. J..

[66] Imai-Nishiya H., Mori K., Inoue M., Wakitani M., Lida S., Shitara K., Satoh M. (2007). Double knockdown of alpha 1,6-fucosyltransferase (FUT8) and GDP-mannose 4,6-dehydratase (GMD) in antibody-producing cells: A new strategy for generating fully non-fucosylated therapeutic antibodies with enhanced ADCC. BMC Biotechnol..

[67] Giddens J.P., Wang L.-X. (2015). Chemoenzymatic Glyco-engineering of monoclonal antibodies. Methods Mol. Biol..

[68] Kurogochi M., Mori M., Osumi K., Tojino M., Sugawara S.-I., Takashima S., Hirose Y., Tsukimura W., Mizuno M., Amano J. (2015). Glycoengineered monoclonal antibodies with homogeneous glycan (M3, G0, G2, and A2) using a chemoenzymatic approach have different affinities for FcγRIIIa and variable antibody-dependent cellular cytotoxicity activities. PLoS ONE.

[69] Umekawa M., Huang W., Li B., Fujita K., Ashida H., Wang L.-X., Yamamoto K. (2007). Mutants of mucor hiemalis endo-β-N-acetylglucosaminidase show enhanced transglycosylation and glycosynthase-like activities. J. Biol. Chem..

[70] Li T., Tong X., Yang Q., Giddens J.P., Wang L.-X. (2016). Glycosynthase mutants of endoglycosidase S2 show potent transglycosylation activity and remarkably relaxed substrate specificity for antibody glycosylation remodeling. J. Biol. Chem..

[71] Wang L.-X., Tong X., Li C., Giddens J.P., Li T. (2019). Glycoengineering of Antibodies for Modulating Functions. Annu. Rev. Biochem..

[72] Bumbaca D., Boswell C.A., Fielder P.J., Khawli L.A. (2012). Physiochemical and biochemical factors influencing the pharmacokinetics of antibody therapeutics. AAPS J..

[73] Jefferis R. (2009). Glycosylation as a strategy to improve antibody based therapeutics. Nat. Rev. Drug Discov..

[74] Smith A., Manoli H., Jaw S., Frutoz K., Epstein A.L., Theil F.P., Khawli L.A. (2016). Unraveling the effect of immunogenicity response on the PK/PD, efficacy, and safety of biologics. J. Immunol. Res..

[75] Lu Y., Khawli L.A., Purushothama S., Theil F.P., Partridge M. (2016). Recent advances in assessing immunogenicity of therapeutic proteins: Impact on biotherapeutic development. J. Immunol. Res..

[76] Beck A., Liu H. (2019). Macro-and micro-heterogeneity of natural and recombinant IgG antibodies. Antibodies.

[77] Reusch D., Tejada M.L. (2015). Fc glycans of therapeutic antibodies as critical quality attributes. Glycobiology.

[78] Duivelshof B.L., Jiskoot W., Beck A., Veuthey J.-L., Guillarme D., D’Atri V. (2019). Glycosylation of biosimilars: Recent advances in analytical characterization and clinical implications. Anal. Chim. Acta.

